# High applicability of two-dimensional phosphorous in Kagome lattice predicted from first-principles calculations

**DOI:** 10.1038/srep23151

**Published:** 2016-03-16

**Authors:** Peng-Jen Chen, Horng-Tay Jeng

**Affiliations:** 1 Department of Physics, National Taiwan University, Taipei 10617, Taiwan; 2 Nano Science and Technology Program, Taiwan International Graduate Program, Academia Sinica, Taipei 11529, Taiwan and National Taiwan University, Taipei 10617, Taiwan; 3 Institute of Physics, Academia Sinica, Taipei 11529, Taiwan; 4 Department of Physics, National Tsing Hua University, Hsinchu 30013, Taiwan

## Abstract

A new semiconducting phase of two-dimensional phosphorous in the Kagome lattice is proposed from first-principles calculations. The band gaps of the monolayer (ML) and bulk Kagome phosphorous (Kagome-P) are 2.00 and 1.11 eV, respectively. The magnitude of the band gap is tunable by applying the in-plane strain and/or changing the number of stacking layers. High optical absorption coefficients at the visible light region are predicted for multilayer Kagome-P, indicating potential applications for solar cell devices. The nearly dispersionless top valence band of the ML Kagome-P with high density of states at the Fermi level leads to superconductivity with *T*_*c*_ of ~9 K under the optimal hole doping concentration. We also propose that the Kagome-P can be fabricated through the manipulation of the substrate-induced strain during the process of the sample growth. Our work demonstrates the high applicability of the Kagome-P in the fields of electronics, photovoltaics, and superconductivity.

The investigation of the two-dimensional (2D) materials has been a rapidly growing field of research since the discovery of graphene^1^,^2^. Despite the novel electrical and mechanical properties of graphene, the semimetallic nature limits its applications in the semiconducting and optoelectronic devices. Thus, the search for new 2D semiconductors becomes an emerging field for their applications in various areas such as semiconductor electronics, optoelectronics, and thermoelectrics. The semiconducting transition metal dichalcogenides, such as MoS_2_^3^,^4^,^5^,^6^,^7^, have been intensively studied in the past few years for their applications in electronics. Recently, an alternative 2D semiconductor, black phosphorus (black-P), has gained much attention because of its potential applications in the semiconductor^8^ and thermoelectric^9^,^10^ devices. In addition to the well-known black-P (*α*- phase), several stable 2D phases of phosphorus have been proposed^11^,^12^, which are termed *β*- (blue), *γ*-, and *δ*- phases. Due to the coordination number of phosphorus, all of the phases form a puckered structure^13^. Electronically, they are all semiconducting with tunable band gaps controlled by either applying the in-plane strain or changing the number of stacking layers. The ability of manipulating the band gap widens the applicability of the 2D phosphorus. Besides, the in-plane connection between different phases of phosphorus is also suggested^12^. By wrapping up the in-plane connected 2D phosphorus, several types of phosphorus nanotubes and fullerene structures are proposed^14^.

On top of these pioneering works, we propose a new novel phase of 2D phosphorus in which the P atoms form the Kagome lattice^15^. This new Kagome phosphorus (Kagome-P) is also a 2D semiconductor. The magnitude of the band gaps, corrected by the hybrid functional of Heyd, Scuseria, and Ernzerhof (HSE)^16^,^17^, ranges from 2.00 eV in monolayer (ML) to 1.11 eV in bulk. The band gap is tunable by applying the in-plane strain and/or changing the number of stacking layers. In addition, our calculations of the optical properties reveal that the absorption coefficients of the visible light are of the order of 10^5 ^cm^−1^. This is comparable with those of the widely used solar cell materials such as GaAs^18^, CdTe^19^, and InP^20^, indicating the potential applications of the Kagome-P in photovoltaic devices. A flat top valance band is found in Kagome-P, yielding high density of states (DOS) right below the Fermi level. As a result, superconductivity takes place in the hole-doped (h-doped) Kagome-P. From our electron-phonon (e-ph) calculations, the *T*_*c*_ can reach ~9 K at the doping level (*n*_*d*_) = 0.15 holes per unit cell (~5.7 × 10^13^ cm^−2^). We also propose that the Kagome-P can be fabricated by applying a compressive in-plane strain to drive the growth mode from the blue phosphorus (blue-P) to the Kagome-P due to the reversed order of the ground state total energies under this condition. This structural phase transition is presumably due to the softer phonons of the Kagome-P that give rise to lower energy costs when compressed laterally. This fact provides an important route toward the experimental realization of the new Kagome-P phase.

## Results and Discussion

**Figure 1:**
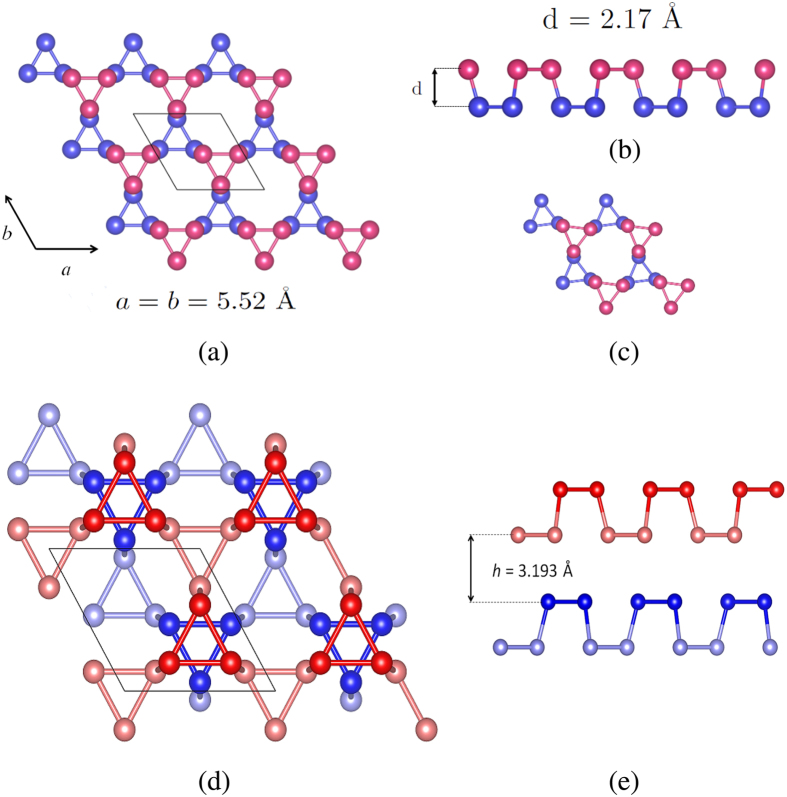
(**a**) Top view and (**b**) side view along **b** direction of the strain-free ML Kagome-P. All the P-P bond lengths in the strain-free ML Kagome-P are 2.25 Å. The upper and lower triangles are represented in different colors for visualization purpose. (**c**) Top view of the distorted ML Kagome-P under compression. (**d**) Top view and (**e**) side view of the AB stacking BL Kagome-P. In (**e**) two different kind of.

The crystal structure of the ML and bilayer (BL) Kagome-P is shown in Fig. 1. The unit cell comprises of an upper and a lower triangles (or sublattices) represented in blue and pink, respectively, in Fig. 1(a–c). The two sublattices are connected by a tilted vertical bond. The relaxed lattice constant is 5.52 Å. The lengths of both the horizontal and the tilted vertical P-P bonds are about 2.25 Å and the buckling distance is 2.17 Å. These values are close to those of phosphorous in other 2D phases. The stability of the Kagome-P, which will be discussed later, is investigated and confirmed by the calculations of the cohesive energy and phonon spectrum.

**Figure 2:**
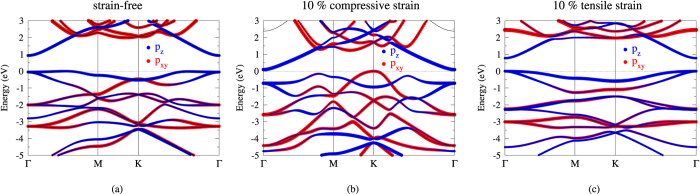
(**a**) Band structure of the strain-free ML Kagome-P. (**b**,**c**) display the band structures under 10% compressive and tensile strain, respectively. The band structures shown here are calculated without the correction of the HSE.

**Figure 3:**
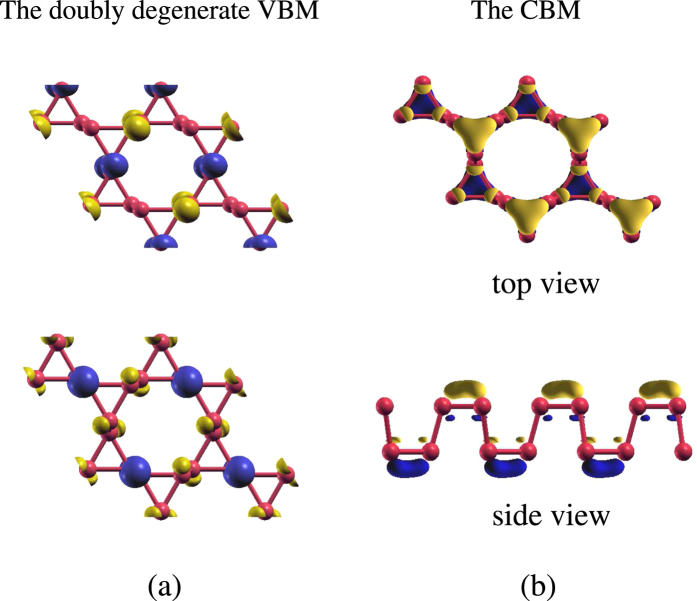
(**a**) The top view of the wave functions of the doubly degenerate states of the top valence band at Γ. (**b**) The wave function of the CBM. The positive and negative values are represented in yellow and blue, respectively.

The DFT calculation reveals that the ML Kagome-P is a semiconductor with an indirect gap of 0.93 eV. As shown in Fig. 2(a), the fairly flat top valence band indicates that the direct (Γ-Γ) band gap assumes nearly the same magnitude. Figure 2(b,c) display the band structures under the 10% compressive and tensile strain, respectively. The effect of the strain will be discussed later. The distinct effective masses of the valence and conduction bands can be explained by the spatial distribution of the wave functions, shown in Fig. 3 for the valence band maximum (VBM) and the conduction band minimum (CBM). We can see from Fig. 3(a) that the localized wave functions of the VBM suppress the inter-site hopping and hence the kinetic energy, leading to the dispersionless valence bands. On the other hand, the graphene-like resonant state of the *p*_*z*_ orbitals within each sublattice, as is displayed in Fig. 3(b), gives rise to a dispersive conduction band.

**Figure 4:**
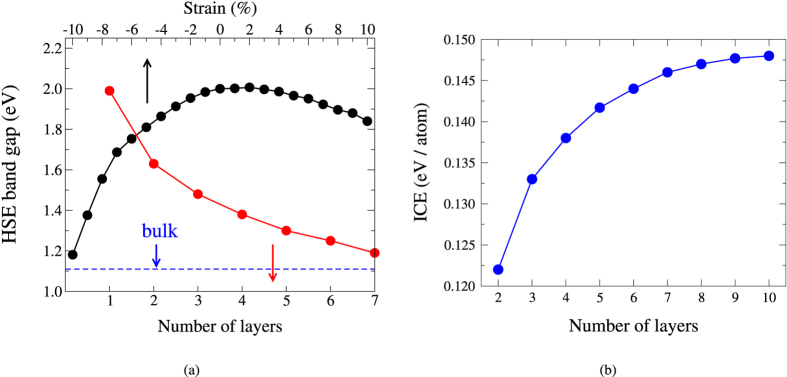
(**a**) Dependence of the HSE-corrected band gaps on the in-plane strain of ML Kagome-P (black dots), and on the number of layers of multilayer Kagome-P (red dots). The gap of the bulk (1.11 eV) is displayed by the blue dotted line. (**b**) The interlayer coupling energy of multilayer Kagome-P.

**Figure 5:**
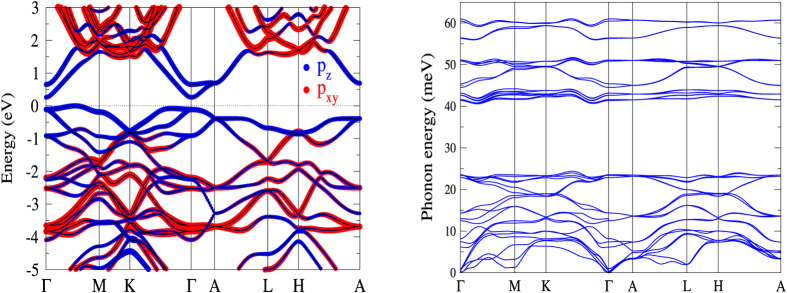
The DFT band structure (**a**) and phonon spectrum (**b**) of the bulk Kagome-P.

Owing to the well-known underestimation of the band gap by DFT, we carry out the HSE calculations implemented in QE to correct the band gaps of the Kagome-P. Like the ML phosphorus in other phases, the band gap of the Kagome-P can also be controlled by the in-plane strain and/or the number of stacking layers. The band gaps of the ML Kagome-P under strain is shown by the black dots in Fig. 4(a). The ML Kagome-P remains semiconducting under the influence of in-plane strain up to 10%. It is found that the tensile strain results in a transition from indirect to direct band gap. We also note that the Kagome-P, when compressed, undergoes a lattice distortion with the phosphorus “triangles” being slightly twisted as shown in Fig. 1(c). In the study of the multilayer and bulk Kagome-P, the way of stacking is an important issue that needs to be clarified. Shown in Fig. 1(d) are the structures of the AB-stacked BL Kagome-P. Different from the blue-P that prefers the ABC stacking, the AB stacking is the most energetically favored structure for multilayer and bulk Kagome-P. Unless otherwise mentioned, the AB stacking is used in the study of the multilayered structure and bulk hereafter. The red dots in Fig. 4(a) represent the dependence of the HSE band gap on the number of layers. The magnitude of the band gap ranges from 2.00 eV in ML to 1.11 eV in bulk. The possibly smallest gap of 0.84 eV could be reached in bulk with ABC stacking. For the ML and bulk we also carry out the GW calculations^21^,^22^ and obtain the gaps of 2.29 eV and 1.28 eV, respectively. The GW approximation increases the HSE-gap by 0.2 ~ 0.3 eV. Taken together the underestimated values as compared with the GW result and the possible overestimate due to the neglect of the excitonic effect, the HSE gaps, which are fundamental gaps, shown in Fig. 4(a) may serve as a good estimate of the measured optical gaps. The interlayer coupling energies (ICEs) of the multilayer Kagome-P, listed in Fig. 4(b), are also investigated to address the stability of the multilayer Kagome-P. The ICE of the Kagome-P is approximately the same with that of the black-P^12^, indicating the stability of the interlayer coupling of the Kagome-P. We also find that the interlayer distance (3.185 ± 0.008 Å) and the in-plane P-P bond length (2.247 ± 0.008 Å) exhibit no significant dependence on the thickness from the ML to bulk. The lattice constant shrinks a little from 5.52 Å in the ML to 5.48 Å in bulk. The band structure and the phonon spectrum of bulk Kagome-P are presented in Fig. 5. It is found that the top valence band in bulk becomes more dispersive than that in the ML, which may be attributed to the strong interlayer coupling.

Figure 4(a) also displays the dependence of band gap on the number of stacking layers (red dots). The band gap decreases due to the interlayer coupling as the number of stacking layer increases. As Fig. 4(a) shows, the strain-free Kagome-P is a semiconductor regardless of the number of layers. It is noted that the band gap decreases under the influence of both the compressive and tensile strains. The reason for this seemingly counterintuitive behavior is as follows. When the lattice is compressed, the *p*_*xy*_ states are pushed upward and replace the *p*_*z*_ states to be the VBM, as Fig. 2(b) depicts. The original nearly dispersionless band along Γ-*M* also shows an increase in energy when hybridizing with the *p*_*xy*_. As a result, the band gap decreases under the compressive strain. The tensile strain, on the other hand, does not show a monotonic effect on the band gap. A small tensile strain (≤2%) slightly increases the band gap. The band gap starts to decrease as the tensile strain becomes larger than 2%, which is also the critical point that the transition from indirect to direct gap takes place. This peculiar behavior is ascribed to the energy shift of the CBM shown in Fig. 2(c). Mediated by the tilting angle of the bond connecting the lower and upper sublattices, all P-P bond lengths remain nearly unchanged when the lattice is stretched. The increased lattice constant elongates the distance between the sublattices, which in turn results in the downshift of CBM due to the reduced Coulomb interactions. Therefore, the band gap becomes smaller under the tensile strain. Similar results can be found in the work done by Yu *et al*.^15^, in spite of some minor difference in the magnitude of band gap that probably stems from different parameters used in the calculations.

In addition to the tunable gap, we also find that the multilayer Kagome-P may possess applicability in solar cell devices. Generally, materials that are considered suitable for the application in solar cells should meet the following two requirements: high absorption coefficients at the visible light wavelengths and appropriate band gap of 1.2 ~ 1.5 eV. As apparently shown in Fig. 4(a), the HSE-corrected electronic band gaps fulfill the required value for solar cell devices. For this reason, we carry out the calculations of the optical properties of the multilayer Kagome-P. Once the dielectric function 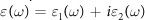 is calculated, the absorption coefficient *α*(*ω*) can then be obtained through the following expression


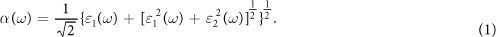


**Figure 6:**
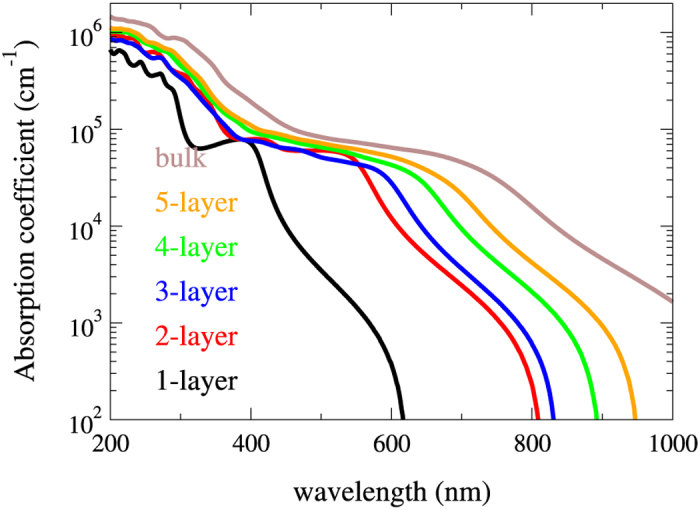
Optical absorption coefficients of the multilayer and bulk Kagome-P. The multilayer Kagome-P exhibits high absorption coefficients within the range of the visible light (400 ~ 700 nm). The HSE-corrected band gaps are adopted here.

The estimated absorption coefficients of the multilayer Kagome-P of different layers are shown in Fig. 6. Comparable with the widely used solar cell materials, the multilayer Kagome-P reveals high absorption coefficients of the visible light of about 10^5^ cm^−1^. The ML Kagome-P, due to the large band gap, fails to reveal suitable absorption coefficients for solar cell devices. The reduced magnitude of the band gap in the multilayer Kagome-P, on the other hand, shifts the high absorption coefficients to the visible light regime. The number of layers, or thickness, of the multilayer Kagome-P has insignificant influence on the absorption coefficients in the visible light regime. This can be understood by the presumably dominant role played by the intra-layer transitions due to the weak interlayer coupling. Nonetheless, the thickness is expected to affect the photoresponsivity that depends on the magnitude of band gap. It is believed that the suitable gap for high optical absorption efficiency ranges from 1.2 to 1.5 eV. Accordingly, the HSE-corrected band gaps shown in Fig. 4 reveal that the few-layer (3 ~ 7) Kagome-P would have the best performance in solar cell devices. Our work demonstrates the high potential of the multilayer Kagome-P in the rapidly growing photovoltaic industry.

**Figure 7:**
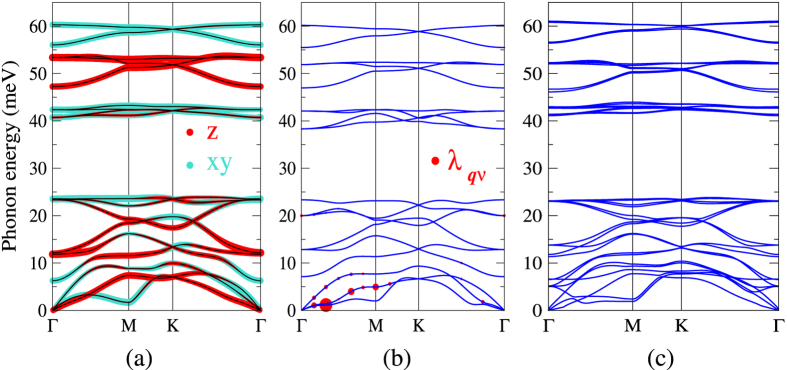
(**a**) Phonon spectrum of the undoped ML Kagome-P with decomposition of the atomic vibrations. The in-plane and out-of-plane atomic motions are displayed in blue and red dots, respectively. (**b**) Phonon spectrum of the h-doped (0.15 holes per unit cell) ML Kagome-P. The size of the red dots represents the strength of the e-ph coupling *λ*_**q***ν*_ of mode *ν* with momentum **q**. (**c**) Phonon spectrum of the AB-stacking bilayer Kagome-P.

The phonon spectra of the bulk and the layered Kagome-P are shown in Figs 5(b) and 7, respectively. These phonon spectra provide direct evidence for the stability of all the Kagome-P studied in this work. Noticeably, the phonon modes are, due to the large phononic band gap of ~17 meV, grouped into two regions: low energy modes with energies up to ~25 meV and high energy modes with energies ranging from 40 ~ 60 meV. More interestingly, the phonon modes that involve in-plane atomic vibrations are nearly dispersionless. These impurity-like characteristics may give rise to phonon localization that blocks the transport of heat^23^. Compared with the black-P that has been reported to be a high *ZT* (~2) material^10^, the few-layer Kagome-P is likely to have a smaller lattice thermal conductivity that is advantageous for high thermoelectric performance. Therefore, the few-layer Kagome-P might also be a good thermoelectric material. The bulk Kagome-P, on the other hand, is on the verge of structural instability as Fig. 5(b) depicts. We would like to mention that the real phonon energies may be higher due to the anharmonicity that is not considered in the calculations.

As mentioned previously, the high DOS around the Fermi level due to the nearly dispersionless top valence band implies the likelihood of exhibiting superconductivity when h-doped. To verify this, we carry out the e-ph calculations, based on the density functional perturbation theory^24^, to evaluate the *T*_*c*_ of the h-doped ML Kagome-P. As listed in Table 1, the increasing DOS with the doping level enhances the e-ph coupling strength. The *T*_*c*_ of the Kagome-P under ambient pressure can achieve 9.12 K at the optimal doping level of 0.15 hole per unit cell. This value is comparable with the *T*_*c*_ of 9.5 K observed in the bulk black phosphorus under the pressure of 32 GPa^25^. It is also expected that *T*_*c*_ would depend on the strain and the number of layers through the change in the DOS. Figure 7(b) displays the phonon spectrum with the optimal h-doping *n*_*d*_ = 0.15 or, equivalently, with carrier concentration of 5.7 × 10^13^ cm^−2^. The e-ph coupling strength λ_**q***ν*_ of mode *ν* with phonon momentum **q** is represented by the size of the red dots. As shown in Fig. 7(b), strong e-ph couplings are originated from the low energy phonon modes along Γ-*M*. Further increasing the doping concentration would lead to the charge density wave instability.

**Figure 8:**
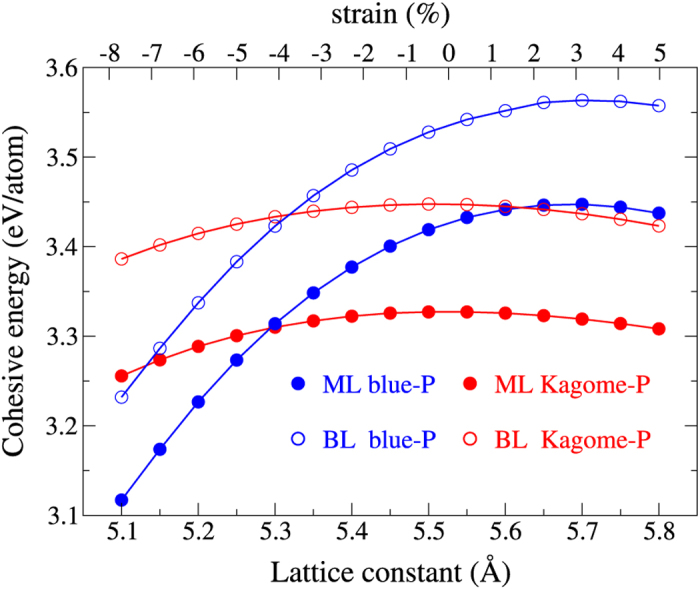
Cohesive energies versus lattice constants of the Kagome-P (red dots) and the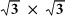 blue-P (blue dots) in ML (solid dots) and BL (open dots) cases. As the figure dictates, the Kagome-P becomes energetically more stable than the blue-P when the lattice constant shrinks below ~5.30 Å. The upper axis label represents the strain with respect to the ML Kagome-P.

The Kagome-P is predicted to exhibit high applicability in many areas. The next issue that we are concerned about is the experimental realization. The calculated cohesive energies of the ML phosphorus in all reported phases are listed in Table 2. The cohesive energy of Kagome-P is 3.33 eV/atom, which is close to those of the *δ*- and *γ*-phosphorus. The Kagome-P is theoretically confirmed to be stable, however, its slightly lower cohesive energy as compared to other phases of 2D phosphorus may cast uncertainty in the fabrication. For this reason, we propose a way to make the fabrication of the Kagome-P feasible. Note that the cohesive energies listed in Table 2 are calculated in the strain-free situation. During the process of the sample growth, strain from the substrate due to lattice mismatch is (and can be made to be) present. This provides a means to selectively grow the desired sample. The calculated cohesive energies of the blue-P and Kagome-P under strain are shown in Fig. 8. It can be seen that both the ML and BL Kagome-P become the favored phase of the 2D phosphorus (in hexagonal lattice) as the lattice constant shrinks below ~5.30 Å. This can be viewed as the consequence of the softer phonons in Kagome-P, which leads to smaller variations of the energy curve in response to the change of the lattice constants. The trilayer blue-P and Kagome-P show nearly the same crossing point as the BL case. This finding demonstrates the feasibility of growing the Kagome-P by manipulating the substrate-induced strain.

## Conclusions

To conclude, we propose a new semiconducting phase of 2D phosphorus that crystallizes in Kagome lattice. The band gap of the ML Kagome-P is 2.00 eV and is tunable by applying the in-plane strain and/or changing the number of stacking layers. We also provide a feasible way of fabricating the Kagome-P by considering the strain exerted by the substrate. High absorption coefficients at the visible light wavelengths and proper magnitude of the band gap indicate that the multilayer Kagome-P could be a potential candidate for the application in solar cell devices. Moreover, it is also predicted to exhibit superconductivity when h-doped. The *T*_*c*_ can reach ~9 K at the optimal carrier concentration of about 5.7 × 10^13^ cm^−2^. Our study reveals that the Kagome-P has wide applications in many fields such as electronics, photovoltaic devices, and superconductivity.

## Methods

The structural optimization and phonon-related calculations are carried out using QUANTUM ESPRESSO code^26^ with PBE functionals^27^ over the 18 × 18 × 1 k-mesh. A 24 × 24 × 1 k-mesh is used for convergence test in phonon calculations. Due to the dispersionless nature of the bands around the Fermi level, it is found that 18 × 18 × 1 is sufficient to yield converged results. The energy cutoff for the plane wave expansion is 40 Ry in the electronic calculations. For phonon calculations, 80 Ry is used to take care of the soft modes. The van der Waals corrections are included in the calculations of the multilayer and bulk Kagome-P via the DFT-D2 functionals^28^. The GW calculations are performed using the BerkeleyGW code^21^,^22^,^29^. The slab truncation of the long-ranged Coulomb interaction is adopted in the ML case^30^,^31^. The calculations of the optical properties are done by Vienna ab initio Simulation Package (VASP)^32^,^33^ with cutoff energy of 500 eV.

## Additional Information

**How to cite this article**: Chen, P.-J. and Jeng, H.-T. High applicability of two-dimensional phosphorous in Kagome lattice predicted from first-principles calculations. *Sci. Rep.* **6**, 23151; doi: 10.1038/srep23151 (2016).

## Figures and Tables

**Table 1:** Superconductivity-related properties in h-doped ML Kagome-P.

*n*_*d*_ (holes/unit cell)	*N*_*F*_ (states/eV)	*λ*	*T*_*c*_ (K)
0.05	1.96	0.56	2.33
0.10	3.29	0.88	7.69
0.15	4.31	2.10	9.12

*N*_*F*_ denotes the DOS at the Fermi level and *λ* is calculated total e-ph coupling strength.

**Table 2:** Calculated cohesive energies (eV/atom) of the ML phosphorus in different phases.

	black-P	blue-P	*γ*-P	*δ*-P	Kagome-P
ML	3.45	3.45	3.36	3.37	3.33
Bulk^12^	3.30 (3.66)	3.29	3.22	3.22	(3.48)

The isolated spin-polarized P atom is taken as the reference. Bulk properties from ref. 12 are also listed for comparison. For black-P and Kagome-P, values from our work are shown in parenthesis. The experimental value of the bulk black-P is 3.43 eV/atom^34^.
